# Simultaneous development of pneumonitis and autoimmune diabetes secondary to immune checkpoint inhibitor treatment with durvalumab in an advanced small cell lung cancer patient: A case report

**DOI:** 10.1097/MD.0000000000032076

**Published:** 2022-12-02

**Authors:** Yan-Ping Wen, Hai-Wei Xiao, Ju-Hua Yin, Hui-Ru Guo, Meng-Jun Shan, Li-Ping Shen, Ling-Shuang Liu

**Affiliations:** a Department of Oncology, Longhua Hospital, Shanghai University of Traditional Chinese Medicine, Shanghai, China; b Department of Traditional Chinese Medicine, Shenshan Medical Center, Memorial Hospital of Sun Yat-sen University, Shanwei, Guangdong Province, China.

**Keywords:** autoimmune diabetes mellitus, checkpoint inhibitor pneumonitis, durvalumab, immune checkpoint inhibitors, immune-related adverse events

## Abstract

Immune checkpoint inhibitors (ICIs) have been widely used in the treatment of various types of cancers worldwide, which is the most significant breakthrough in cancer therapy in recent years. Despite their excellent benefits in anti-tumor efficacy, a subset of patients will experience various autoimmune toxicities, termed as immune-related adverse events (irAEs), which can affect almost any organ systems, but related to the pulmonary and pancreatic islets simultaneously has rarely been reported and discussed.

**Patient concerns:**

In this report, we describe a rare case of a 65-year-old man patient with advanced small cell lung cancer (SCLC) who suffered general fatigue, dry cough, chest tightness, shortness of breath and polyuria-polydipsia syndrome after the eighth cycle treatment with programmed cell death ligand-1 (PD-L1) inhibitor durvalumab.

**Diagnoses:**

According to the results of laboratory tests, chest computed tomography and multidisciplinary discussion, the patient was eventually diagnosed with ICI-related pneumonitis and autoimmune diabetes mellitus.

**Interventions:**

Multiple daily subcutaneous insulin injections, empirical anti-infection and immunosuppression treatment with corticosteroids were performed.

**Outcomes:**

After the cessation of durvalumab and comprehensive treatment, the patient’s respiratory condition was relieved significantly and his blood glucose was well controlled with insulin therapy.

**Lessons:**

With the widespread use of ICIs, there will be more patients developing these rare but severe irAEs in clinical practice, which should attract great attention of both clinicians and patients.

## 1. Introduction

Immune checkpoint inhibitors (ICIs), such as programmed cell death 1 (PD-1), programmed cell death ligand-1 (PD-L1) and cytotoxic T-lymphocyte antigen-4 (CTLA-4) inhibitors, are the major breakthrough in cancer therapy in recent years, which can greatly improve survival in a subgroup of cancer patients by blocking immunosuppressive molecules and reactivating the function of effector T cells to specifically kill tumor cells.^[1]^ However, excessively activated immune cells may also attack normal tissues and organs, leading to autoimmune injuries in almost any body systems, collectively known as immune-related adverse events (irAEs), most commonly in the gastrointestinal tract, liver, skin and endocrine systems.^[2]^ Several large-scale clinical trials reported that the incidence of any grade irAEs was up to 60% to 80% in patients treated with PD-1/PD-L1 inhibitors monotherapy, of which 7% to 17% were grade 3/4.^[3–5]^ And combination treatment with 2 ICIs appears to rise both the incidence and the severity of irAEs compared to single ICI agent. A large pooled analysis of patients accepting nivolumab plus ipilimumab combination therapy showed that almost all treated patients (94.9%) experienced at least 1 irAE, even more than one-half of patients (55.4%) developed grade 3/4 irAEs.^[6]^

Among the various irAEs, checkpoint inhibitor pneumonitis (CIP) is generally uncommon, with a frequency of 3% to 5% for PD-1/PD-L1 inhibitors and 1% for CTLA-4 inhibitors in clinical trial settings,^[7–9]^ but if not treated timely, it is a potentially fatal or life-threatening complication, accounting for 28% of ICI-induced deaths.^[10]^ However, it should be noted that the incidence of CIP was up to 13% to 19% in real-world practice,^[11,12]^ which is much higher than those observed in clinical studies. ICI-mediated endocrinopathies are among the most frequent irAEs, which occur in 4% to 30% of patients,^[13]^ while ICI-related autoimmune diabetes mellitus (DM) is extremely rare, with an observed incidence ranging from 0.2% in randomized clinical trials^[14]^ to 0.9% in real-world settings.^[15]^ Herein, we report an especially rare case of an advanced small cell lung cancer (SCLC) patient who simultaneously developed CIP and DM after the eighth cycle treatment with PD-L1 inhibitor durvalumab.

## 2. Case report

In September 2019, a 65-year-old Chinese man with history of former tobacco use (12 pack-years) was diagnosed with compound small cell carcinoma (containing squamous cell carcinoma component) of left lower lobe of lung [cT2aN3M1a, stage IVA (AJCC 8^th^ edition)] without tumor driver gene alterations, the PD-L1 tumor proportion score (TPS) was unknown. He had a history of coronary heart disease, hypertension, lacunar cerebral infarction, and denied personal or family history of DM or autoimmune diseases.

**Figure 1.**
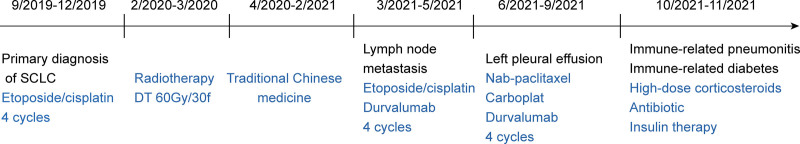
Time axis of the patient’s anti-tumor treatment and intervention on immune-related pneumonitis and diabetes mellitus. SCLC = small cell lung cancer.

**Figure 2.**
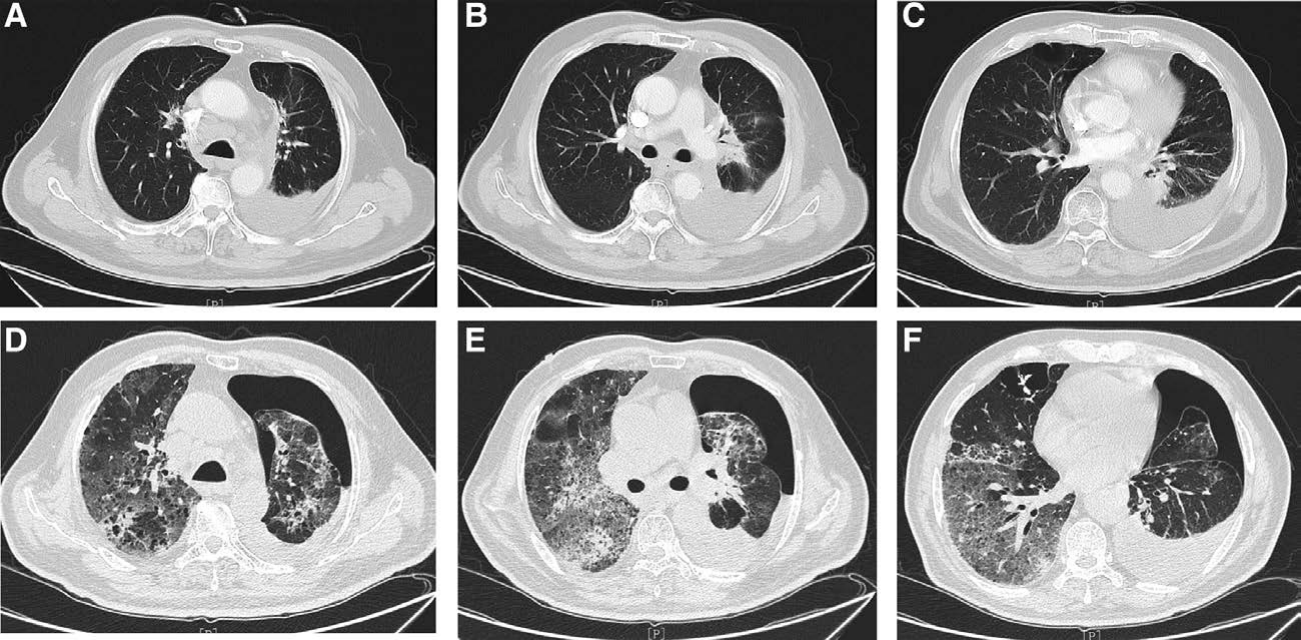
Immune-related pneumonitis. (A–C) After the fourth course of chemotherapy + durvalumab treatment, a chest CT scan showed no obvious inflammatory lesions in the lungs. (D–F) Chest CT scan on admission (5 weeks after the eighth course of chemotherapy + durvalumab treatment) showed non-segmentally distributed extensive consolidations and GGOs in the bilateral lungs. CT = computed tomography, GGOs = ground-glass opacities.

From October 2019 to March 2020, the patient received 4 cycles of systemic chemotherapy consisting of etoposide (100 mg day 1–5, every 3 weeks) and cisplatin (80 mg d1, 70 mg d2, every 3 weeks), followed by thoracic radiotherapy (60 Gy/30 f) as a first-line treatment (Fig. 1). During this period, the best effect was assessed as partial response (PR) according to Response Evaluation Criteria in Solid Tumors version 1.1 (RECIST v1.1), without any evidence of disease progression. Since April 2020, he was treated with traditional Chinese medicine (TCM) alone for nearly 1 year, which did improve his quality of life significantly and get his disease controlled. In March 2021, a computed tomography (CT) scan of chest showed a progression of disease, therefore, he was administered durvalumab (1000 mg d1) in combination with etoposide/cisplatin chemotherapy every 3 weeks as a second-line therapy. After 4-cycle treatment of this regimen, chest CT suggested that the tumor lesions were stable but left pleural effusion increased, and no obvious inflammatory lesions were observed in the bilateral lungs (Fig. 2A–C). Subsequently, he commenced a third-line treatment with nab-paclitaxel (300 mg d1, 200 mg d8), carboplatin (600 mg d1) and durvalumab (1000 mg d1) in a 21-day cycle, considering the presence of squamous cell carcinoma. Four courses treatment later, the response was considered as stable disease.

In the end of October 2021, 5 weeks after the eighth cycle of durvalumab and chemotherapy, he was admitted to the hospital with a 7-day history of general fatigue, dry cough, chest tightness and shortness of breath. The admission physical examination revealed a temperature of 37.9°C, heart rate of 103 bpm, blood pressure of 132/89 mm Hg, respiration of 26 per minute with oxygen saturation (SpO2) of 88% at room air, bilateral attenuated breathing sound and right wet rale were auscultated, and the remainder of examination was unremarkable. Initial laboratory tests showed white blood cell (WBC) of 12.60 × 10^9^/L, hypersensitive C-reactive protein (hs-CRP) of 130.01 mg/L, K of 3.4 mmol/L, PaO2 of 57 mm Hg, SaO2 of 93%, B-type natriuretic peptide (BNP) of 126 pg/mL (no obvious change from his baseline value), and his left ventricular ejection fraction examined by ultrasound cardiography was 62%. Therefore, involvement of congested heart failure was ruled out. Empirical anti-infective with levofloxacin and oxygen therapy were initially administrated. Four days later, the temperature was normal, but the patient’s tachypnea was aggravated, and accompanying new-onset polyuria-polydipsia syndrome. Further laboratory workup revealed a markedly elevated WBC of 23.64 × 10^9^/L, hs-CRP of 21.95 mg/L, blood glucose level of 17.2 mmol/L, glycated hemoglobin (HbA1c) level of 7.9%, pH of 7.430, PaO2 of 59 mm Hg, SaO2 of 91%, bicarbonate of 25.9 mmol/L. Glucose and ketones in urine were all negative (Table 1). Blood and sputum culture did not suggest any causative microbial organism. Meanwhile, the patient received a chest CT scan which demonstrated that non-segmentally distributed extensive consolidations and ground-glass opacities (GGOs) in the bilateral lungs, pleural effusion and pneumothorax in the left lung (Fig. 2D–F). After multidisciplinary discussion, including a respiratory specialist, endocrinologist, and thoracic surgeon, the diagnosis of durvalumab-induced pneumonitis (CTCAE-grade 3) and dubious autoimmune diabetes (CTCAE-grade 3) were considered. Thus, closed thoracic drainage, multiple daily subcutaneous insulin injections and intravenous methylprednisolone (40 mg, twice a day) were immediately performed. In addition, imipenem and cilastatin sodium (1 g, once every 12 hours) in combination with levofloxacin (0.5 g, once a day) were given to prevent infection for 7 days. After the cessation of durvalumab and comprehensive treatment, the patient’s symptoms were obviously alleviated and abnormal indications gradually improved. Therefore, the dose of methylprednisolone was decreased to 40 mg/d in a week, and then replaced by oral prednisone and tapered over 1 month. Encouragingly, his blood glucose was controlled well with insulin therapy during corticosteroid treatment. Unfortunately, the patient refused a chest CT reexamination, so we were unable to obtain imaging data for comparison before and after steroid treatment.

**Table 1:** Laboratory results on the fifth week after the eighth administration of durvalumab.

Test	Value	Reference	Test	Value	Reference
Blood			Arterial blood gas		
WBC (×10^9^/L)	23.64	3.5–9.5	PH	7.430	7.35–7.45
Neutrophils (%)	92.3	40–75	PaO2 (mm Hg)	59	83–108
Lymphocytes (%)	3.2	20–50	PaCO2 (mm Hg)	39	35–48
Hemoglobin (g/L)	77	130–175	SaO2 (%)	91	95–98
Platelet (×10^9^/L)	187	125–350	HCO3^−^ (mmol/L)	25.9	22–26
hs-CRP (mg/L)	21.95	0–5	BE (mmol/L)	1.5	−2 to 3
Procalcitonin (ng/mL)	0.06	<0.5	Lactic acid (mmol/L)	2.9	0.5–1.6
Glucose (mmol/L)	17.2	3.9–6.1	Urinalysis		
HbA1c (%)	7.9	3.6–6	Glucose	Negative	Negative
ALT (U/L)	105	13–69	Ketones	Negative	Negative
AST (U/L)	46	15–46			
Creatinine (μmol/L)	40.9	28–110			
BNP (pg/mL)	124	0–100			
Na (mmol/L)	139.3	137–145			
K (mmol/L)	4.6	3.5–5.1			
Cl (mmol/L)	104.9	98–107			
Ca (mmol/L)	2.11	2.1–2.55			
P (mmol/L)	1.16	0.81–1.45			

ALT = alanine aminotransferase, AST = aspartate aminotransferase, BE = base excess, BNP = B-type natriuretic peptide, HbA1c = glycated hemoglobin, HCO3- = bicarbonate ion, hs-CRP = hypersensitive C-reactive protein, WBC = white blood cell.

Due to the seriousness of irAEs and poor physical condition of this patient, an ICI re-challenge therapy was not administered. TCM was selected as the subsequent anti-tumor treatment and his glycemic control still depended on insulin.

## 3. Discussion

With the widespread application of ICIs worldwide, irAEs have become increasingly common, even more and more uncommon irAEs are also being reported. IrAEs can affect almost any organ systems, but it related to the pulmonary and pancreatic islets simultaneously has rarely been reported and discussed. To the best of our knowledge, this is an extremely rare case report that describes the simultaneous development of pneumonitis and DM after 8 cycles treatment with PD-L1 inhibitor (durvalumab) in an advanced SCLC patient.

ICI-related pneumonitis (CIP) is a relatively uncommon but clinically important and potentially life-threatening adverse event. It can occur at any time during or after ICI therapy, the median (range) times from initiation of ICI treatment to the development of pneumonitis were 2.8 (0.3–19.2) months as reported by Naidoo et al,^[7]^ 2.6 (0.5–11.5) months as reported by Nishino et al,^[16]^ and 2.3 (0.2–27.4) months as reported by Delaunay et al.^[17]^ These studies also demonstrated that CIP tended to occur earlier in lung cancer patients than those with other type cancer, higher pulmonary tumor burden resulting in earlier onset of respiratory symptoms may explain this phenomenon. In our case, the interval was 7 months, which is later than the median times in previous reports. But Delaunay et al reported that the median time to onset and the severity of pneumonitis had no relevant correlation.^[17]^ The clinical manifestation of CIP is nonspecific, ranging from asymptomatic disease to cough, dyspnea, fever, chest pain, and may present with hypoxia and respiratory distress in some severe cases.^[7]^ The chest CT findings of CIP are also nonspecific, in contrast, it showed a wide spectrum of radiological presentation. The most common radiographic pattern is cryptogenic organizing pneumonia (COP), followed by hypersensitivity pneumonia (HP), nonspecific interstitial pneumonia (NSIP), and acute interstitial pneumonia (AIP)/acute respiratory distress syndrome (ARDS).^[12,16,17]^ Notably, these radiographic patterns are associated with the toxicity grades of CIP, AIP/ARDS pattern had the highest grade, followed by COP, while NSIP and HP had lower grade.^[16]^ The results indicate the value of the radiographic pattern-based method in categorizing and prognosticating CIP.

Lack of specificity in the clinical and radiological presentations poses a huge challenge to clinical diagnosis of CIP. In fact, it is typically a diagnosis of exclusion, which requires ruling out all types of pulmonary infections, tumor progression or radiation-induced pneumonitis. A bronchoscopic examination of the bronchoalveolar lavage fluid and a lung biopsy can be useful for diagnosing CIP. Although bronchoscopy and lung biopsy were not performed due to the patient’s incompatibility, laboratory and imaging examination were also able to eliminate the involvement of infectious pulmonary inflammation and tumor progression in present case. In the setting of ICI therapy and prior thoracic radiotherapy, CIP must be differentiated from radiation pneumonitis (RP). Generally, RP develops 2 to 4 months after radiotherapy, the lesion area is limited to the radioactive field, and the imaging features are interstitial changes with regular borders.^[18,19]^ In our case, the patient had a twenty-month history of radiotherapy, and his chest CT showed a non-segmentally distributed extensive consolidations and GGOs in the bilateral lungs. Based on these 2 points, we did not consider the diagnosis of RP. What’s more, studies have shown that the patients with lung cancer, prior lung disease (especially interstitial lung disease), prior thoracic radiotherapy, or combination therapy with ICIs or other drugs have a relative high risk to develop CIP than those without. Additionally, smoking history, an age older than 70 years, and a poor performance status are all potential risk factors of CIP.^[20,21]^ Therefore, in clinical practice, we should attach close attentions to these patients who have risk factors, in order to facilitate an early recognition and intervention of high-risk groups.

ICI-induced autoimmune DM is a rare but often life-threatening metabolic urgency, which is considered to be a distinct diabetes subtype, showing typical characteristics of classic type 1 diabetes mellitus (T1DM) and fulminant diabetes. The rapid-onset of hyperglycemia accompanied with a high incidence of diabetic ketoacidosis (DKA), relatively low HbA1c levels and undetectable serum C-peptide are suggestive of fulminant diabetes, while the presence of islet autoantibodies and susceptible human leukocyte antigen (HLA) genotypes fit to the classic T1DM. In a systematic review of autoimmune DM secondary to ICIs, 91 cases worldwide were analyzed.^[22]^ In that analysis, 71% of patients presented with DKA at primary onset, with a median plasma glucose of 31.4 mmol/l (range, 11.6-67.3), a HbA1c levels of 7.6% (range, 5.4–11.4), and 84% of these cases showed a low or undetectable C-peptide levels. Pancreatic islet autoantibodies were detected in 53% of patients, with a majority of glutamic acid decarboxylase antibody (GADA),^[22]^ which suggested that the genesis of autoimmune diabetes induced by ICI therapy was not exactly same as classic T1DM where autoantibodies were present in 80% to 95% of patients.^[23]^ Of note, several previous studies revealed that the presence of islet autoantibodies at the time of diagnosis is related to an earlier onset of diabetes,^[22,24,25]^ just as the systemic review reported, the mean time of onset was 3.1 cycles for GADA-positive and 5.9 cycles for GADA-negative patients.^[22]^ The susceptible HLA genotypes were present in 61% cases and the HLA-DR4, DR3, DR9, and A2 were the predominant HLA serotypes.^[22]^ Another retrospective study by Stamatouli et al also revealed that 76% of patients with ICI-induced DM showed a predominance of HLA-DR4.^[15]^ Thus, we infer that the presence of islet autoantibodies and susceptible HLA genotypes at baseline may be potential risk factors for the development of DM related to ICI. Nevertheless, these 2 parameters were rarely tested in patients since they are not recommended in routine clinical practice, their effectiveness and economic benefits as predictive biomarkers for DM need more validation in future studies.

**Figure 3.**
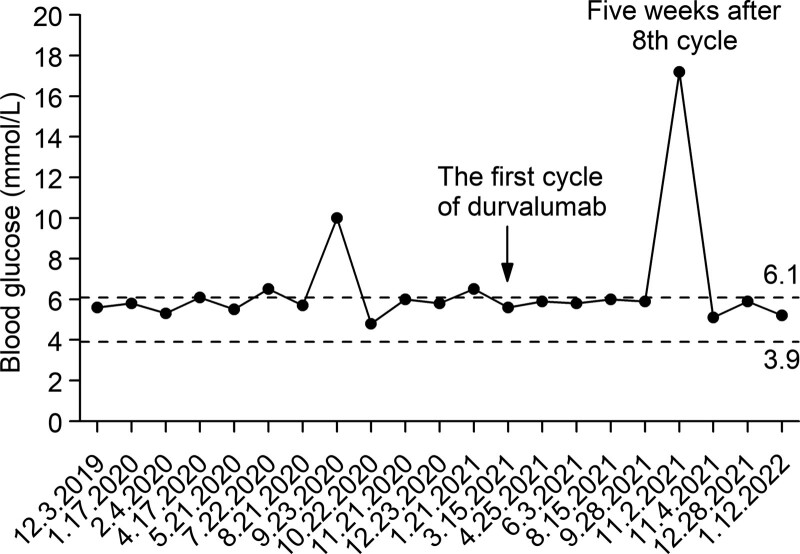
The patient’s blood glucose level. Two dotted lines indicate the upper and lower limit of normal blood glucose. Before November 2, 2021, the patient never used hypoglycemic drugs, after this time, his glycemic control depended on insulin.

Actually, our patient received routine monitoring of blood glucose, showing a basically normal level (never using hypoglycemic drugs), regardless several abnormal blood glucose values were observed before ICI treatment (Fig. 3). But on the fifth week after 8 cycles of durvalumab, he developed severe CIP accompanying with polyuria-polydipsia syndrome, and his blood glucose abruptly increased to 17.2 mmol/L with a relatively low HbA1c levels (7.9%). Then, steroid and insulin therapy were administrated together, interestingly, after the mitigation of CIP and cessation of steroids, his blood glucose did not return to baseline and glycemic control still depended on insulin. Although neither serum C-peptide nor islet autoantibodies were assessed, autoimmune DM related to durvalumab therapy was strongly suspected, because there were no other factors associated with glucose metabolism. The luck of present case is that the timely recognition and treatment of glycemic abnormality avoided the development of DKA, a potentially lethal condition.

Several guidelines or consensus for the management and treatment of irAEs have been published,^[26–28]^ which all recommend corticosteroids as the main therapeutic modality for CIP. It has been previously reported that CIP can be sufficiently managed by drug withdrawal and steroid therapy in majority of patients.^[7,17]^ However, in clinical practice, there are a subset of patients with steroid-refractory CIP cannot improve initially or completely, and additional immunosuppressive agents such as infliximab, cyclophosphamide, intravenous immunoglobulin, or mycophenolate mofetil should be considered.^[26]^ Unlike most non-endocrine irAEs that are responsive to steroid therapy, ICI-induced DM is not reversed by high-dose corticosteroids and requires long-term insulin therapy since most of the β cells function are irreversibly destroyed.^[26–28]^ Some clinicians tried to use glucocorticoids to salvage β cells function but disappointingly, was unsuccessful, inversely, insulin resistance was seen in some patients.^[29,30]^ Fortunately, our patient got a good clinical response both in CIP and hyperglycemia with corticosteroid and insulin therapy simultaneously. As there are no practically available predictive biomarkers for the occurrence of DKA or diabetes and the onset time is also difficult to predict, ranging from 5 to 790 days after the first cycle of ICIs,^[31]^ even some patients developed DM 4 months or more after stopping ICI therapy.^[32,33]^ Therefore, routine monitoring of blood glucose before each administration of ICIs is required in all patients regardless of the previous history of diabetes, which would theoretically allow for an early recognition of glucose abnormalities.

## 4. Conclusion

We showed a case of anti PD-L1 monoclonal antibody induced pneumonitis and DM simultaneously in SCLC treatment. These 2 adverse events are both uncommon but life-threatening if not recognized and treated timely. Moreover, with the widespread use of ICIs in various cancer types, there will be more patients developing these rare and severe irAEs in clinical practice, which should attract attention of both clinicians and patients. Clinicians should thoroughly evaluate the potential risk of irAEs before ICI administration, carefully observe related signs and symptoms during ICI treatment, and even follow-up at least half a year after discontinuation of ICI therapy due to a possibility of delayed irAEs. Meanwhile, the patients should be educated to identify and report symptoms as early as possible to their treating physician in order to get a timely medical care. Furthermore, when irAEs is suspicious, a multidisciplinary discussion should be arranged to achieve favorable outcomes.

## Author contributions

All authors have made substantial contributions to the work, and all authors have read and approved the final manuscript.

**Conceptualization:** Yanping Wen, Haiwei Xiao, Ling-Shuang Liu.

**Data curation:** Ju-Hua Yin, Hui-Ru Guo, Meng-Jun Shan.

**Investigation:** Yanping Wen, Li-Ping Shen

**Project administration:** Ling-Shuang Liu.

**Supervision:** Ling-Shuang Liu.

**Writing – original draft:** Yanping Wen, Haiwei Xiao.

**Writing – review & editing:** Yanping Wen, Ju-Hua Yin, Ling-Shuang Liu.
